# Fumonisins (Natural Toxins and Mycotoxins)

**DOI:** 10.14252/foodsafetyfscj.2018006s

**Published:** 2018-12-21

**Authors:** Food Safety Commission of Japan

## Abstract

The Food Safety Commission of Japan (FSCJ) conducted a self-tasking assessment of mycotoxins, fumonisin B1 (FB1 CAS No. 116355-83-0), fumonisin B2 (FB2 CAS No. 116355-84-1), and fumonisin B3 (FB3 CAS No. 136379-59-4). Hepatotoxicity and/or nephrotoxicity were commonly observed in experimental animals given orally purified FB1, and the sex-related differences were observed in rats and mice. Species differences were also identified: Increased incidences of liver tumors in female mice and of kidney tumors in male rats were observed in chronic toxicity/carcinogenicity studies. Fumonisins did not show appreciable genotoxicity both the *in vivo* and *in vitro* tests. FSCJ judged fumonisins as non-genotoxic carcinogens from the results of various toxicological studies on fumonisins, and thus specified a tolerable daily intake (TDI) of 2 μg/mg bw/day for fumonisins (FB1, FB2 and FB3, alone or by combination), after applying an uncertainty factor of 100 to the lowest no-observed-adverse-effect level (NOAEL) of 0.21 mg/kg bw/day in subacute toxicity study in rats. The estimated exposure levels of fumonisins among high consumers such as toddlers are still below the TDI. Therefore, FSCJ concluded that adverse effect of fumonisin on human health through food are unlikely under the current situation in Japan.

## Conclusion in Brief

The Food Safety Commission of Japan (FSCJ) conducted a self-tasking assessment of mycotoxins, fumonisin B1 (FB1 CAS No. 116355-83-0), fumonisin B2 (FB2 CAS No. 116355-84-1), and fumonisin B3 (FB3 CAS No. 136379-59-4).

Fumonisins, produced by fungi of the genus Fusarium such as *F. verticillioides* and *F. proliferatum*, are detected particularly in maize and maize-based products worldwide. Experimental and epidemiological findings suggest that fumonisins cause equine leukoencephalomalacia (ELEM) and porcine pulmonary edema (PPE). Association of intake of fumonisins with human neural tube defects (NTD) in fetus has also been shown in regions where maize is consumed as a major food source.

The data used in the assessment include pharmacokinetics in experimental animals, acute toxicity, subacute toxicity, chronic toxicity/carcinogenicity, reproductive and developmental toxicity, and genotoxicity.

Hepatotoxicity and/or nephrotoxicity were commonly observed in experimental animals given orally purified FB1, and the sex-related differences were observed in rats and mice. Species differences were also identified: Increased incidences of liver tumors in female mice and of kidney tumors in male rats were observed in chronic toxicity/carcinogenicity studies. Fumonisins did not show appreciable genotoxicity both the *in vivo* and *in vitro* tests.

FSCJ judged fumonisins as non-genotoxic carcinogens from the results of various toxicological studies on fumonisins, and thus specified a tolerable daily intake (TDI) of 2 μg/mg bw/day for fumonisins (FB1, FB2 and FB3, alone or by combination), after applying an uncertainty factor of 100 to the lowest no-observed-adverse-effect level (NOAEL) of 0.21 mg/kg bw/day in subacute toxicity study in rats.

The estimated exposure levels of fumonisins among high consumers such as toddlers are still below the TDI. Therefore, FSCJ concluded that adverse effect of fumonisin on human health through food are unlikely under the current situation in Japan.

The climate may affect mycotoxin contamination in crops every harvest year, and thus risk managers should be aware of monitoring fumonisin contamination levels in foods and of introducing the standard as necessary.

For modified fumonisins, only limited scientific findings are available, and therefore, it is necessary to collect continuously the latest scientific findings and information.

